# Overexpression of DTL enhances cell motility and promotes tumor metastasis in cervical adenocarcinoma by inducing RAC1-JNK-FOXO1 axis

**DOI:** 10.1038/s41419-021-04179-5

**Published:** 2021-10-11

**Authors:** Sijia Liu, Lina Gu, Nan Wu, Jiayu Song, Jiazhuo Yan, Shanshan Yang, Yue Feng, Zhao Wang, Le Wang, Yunyan Zhang, Yan Jin

**Affiliations:** 1 Department of Gynecological Radiotherapy, Harbin Medical University Cancer Hospital, Harbin, Heilongjiang 150081 China; 2 Key laboratory of preservation of human genetic resources and disease control in China (Harbin Medical University), Ministry of Education, Harbin, Heilongjiang 150081 China

**Keywords:** Cancer epigenetics, Cervical cancer

## Abstract

Cervical adenocarcinoma is an important disease that affects young women and it has a high mortality and poor prognosis. Denticleless E3 ubiquitin protein ligase homolog (DTL) gene with oncogenic function has been evaluated in several cancers. Through this study, we aimed to clarify the clinical and molecular characteristics of cervical adenocarcinoma involving overexpression of DTL and elucidate its molecular mechanism. Bioinformatics analysis was performed through multiple databases. RNA sequencing was used to obtain differentially expressed genes after DTL was overexpressed in cells. The role of DTL in cervical adenocarcinoma was explored through in vitro and in vivo experiments. We found that DTL has an unfavorable prognostic implication for patients with cervical adenocarcinoma. Overexpression of DTL induced the migration and invasion of tumor cells in vitro and promoted intra-pulmonary metastasis in vivo. In addition, DTL activated JNK through RAC1 and upregulated FOXO1 to induce epithelial–mesenchymal transition, and the migration and invasion of tumor cells. Therefore, we conclude that overexpression of DTL enhanced cell motility and promoted tumor metastasis of cervical adenocarcinoma by regulating the RAC1-JNK-FOXO1 axis. These results suggest that DTL may become a potential therapeutic target for antitumor metastasis of cervical adenocarcinoma.

## Introduction

Cervical cancer is the fourth most common cancer in terms of incidence and mortality among women worldwide [1]. Although long-term extensive screening helps to gradually remove the slow-growing squamous lesions, the proportion of adenocarcinoma continues to increase over time [2]. Several retrospective studies have revealed that patients with cervical adenocarcinoma have a higher risk of developing metastasis, leading to a poorer prognosis [3–5]. However, regardless of the cancer subtype, primary treatment with curative intent for patients with cervical cancer typically consists of surgery, chemoradiation, or a combination of these treatments [6]. Thus, no treatment guidelines for cervical adenocarcinoma have been established and a more effective therapeutic target has not been found. Therefore, there is an urgent need to investigate the mechanisms underlying the metastasis of cervical adenocarcinoma and its related molecular alterations.

Epithelial–mesenchymal transition (EMT) is a developmental process in which the cellular signaling programs in epithelial cells are re-modified and usually promote morphological changes, especially the elongated spindle-like mesenchyme with migration and invasion characteristics [7]. EMT plays an important role in tumor progression and is related to many features of cancer [8]. Tumor cells grow in a mesenchymal shape and show considerably enhanced migration, invasion, and metastasis capabilities [9], particularly those of metastasis. E3 ubiquitin ligase Hakai ubiquitinates E-cadherin, which is involved in the modulation of EMT during progression or metastasis [10]. Denticleless E3 ubiquitin protein ligase (DTL), a homolog of E3 ubiquitin ligase, belongs to the DCAF protein family containing WD40 repeats and plays a biological role as a substrate receptor of CRL4 ubiquitin ligase [11]. Previous studies have shown that DTL is related to the regulation of DNA damage repair, cell cycle, and cell apoptosis, which induces chromosomal separation and cell division defects, and that it is a key regulator of cell cycle progression and genome stability [12, 13]. Research regarding the role of DTL in solid tumors started relatively late and it is noteworthy that no study has reported the correlation between DTL and cervical adenocarcinoma. Moreover, the molecular mechanisms underlying the promotion of progression of EMT, and tumor invasion and metastasis by DTL are still unclear.

Through the current study, we aimed to explore whether DTL-mediated migration and invasion of cancer cells was related to EMT by c-Jun N-terminal kinase (JNK) pathway. We observed that DTL is associated with tumor invasion and has an unfavorable prognostic implication for patients with cervical adenocarcinoma. To investigate the effect of DTL on cervical adenocarcinoma, we constructed DTL-overexpressing cell lines. Overexpression of DTL induced cell migration and invasion. In addition, we confirmed that increased activation of the JNK pathway in DTL-overexpressing cells promoted the expression of FOXO1, which led to EMT. These results further enhance our understanding of the invasion and migration of tumor cells in cervical adenocarcinoma, and suggest that DTL could serve as a potential prognostic marker to predict the prognosis of patients with cervical adenocarcinoma.

## Materials and methods

### Data collection and tissue specimens

Two transcriptome data sets were used for patients diagnosed with cervical adenocarcinoma, namely The Cancer Genome Atlas (TCGA, 30 samples) data set (http://cancergenome.nih.gov/) and the Gene Expression Omnibus (GEO) database (www.ncbi.nlm.nih.gov/geo), including GSE64217, GSE63514, and GSE9750. Specifically, there were 2 normal tissues and 2 cervical cancer tissues in GSE64217, 24 normal tissues and 27 cervical cancer tissues in GSE63514, and 24 normal tissues and 32 cervical cancer tissues in GSE9750. In this study, 135 patients with cervical adenocarcinoma who received surgical treatment at the Department of Gynecology, Harbin Medical University Cancer Hospital (Harbin, China) from 16 August 2010 to 21 December 2016 were recruited. Informed consent was obtained from the patients or their family members before enrollment in the study. The study protocol was approved by the Clinical Research Ethics Committee of Harbin Medical University Cancer Hospital (Harbin, China).

### Cells and reagents

Human cervical cancer Hela cell line was acquired from Shanghai Gaining Biological Technology Co., Ltd (Shanghai, China). Caski cell line was obtained from Beijing Beina Chuanglian Biotechnology Research Institute. SiHa cell line was gifted by Laboratory of Medical Genetics, Harbin Medical University. All cell lines were recently authenticated by Short Tandem Repeat (STR) profiling. SiHa cell lines were grown in Minimum Essential Medium. Hela and Caski cell lines were cultured in Roswell Park Memorial Institute 1640 media. All cell lines were cultured in media with 10% fetal bovine serum (FBS; BD Biosciences, San Jose, CA, USA) at 37 °C in incubators with 5% CO_2_. Cells were passaged at a confluency of 80%.

### Cell transfection

Cells were plated in six-well plates 24 h before transfection. For DTL overexpression, viruses containing the DTL sequence (NM_016448.4, GeneChem, Shanghai, China) were transfected into SiHa and Caski cells for another 12 h, and cells were selected by puromycin treatment for 7 days. DTL overexpression was confirmed by western blotting. For FOXO1, DTL, RAC1, and CDC42 knockdown, small interfering RNAs (siRNAs) targeting FOXO1 (stB0001141), DTL (stB0003394), RAC1 (stB0002555), and CDC42 (stB0002526), acquired from RiboBio (Guangzhou, China), were transfected into SiHa and Caski cells with Lipofectamine 2000 (Invitrogen, Carlsbad, CA, USA).

### Western blotting

Cells were lysed in radioimmunoprecipitation assay buffer (Thermo Fisher Scientific, Waltham, MA, USA) with 1% protease inhibitors (Roche, Basel, Switzerland). Protein extracts were subjected to electrophoresis on 10% SDS-polyacrylamide gel electrophoresis and transferred onto polyvinylidene difluoride membranes (Millipore, Billerica, MA, USA). The immunoblots were sealed with 5% milk-Tris-Buffered Saline with Tween 20 (TBST) solution and incubated separately with rabbit anti-DTL (1:800, Abcam, ab72264), rabbit anti-FOXO1 (1:800, Cell Signaling Technology, #2880), rabbit anti-Phospho-stress-activated protein kinase (SAPK)/JNK (1:800, Cell Signaling Technology, #4668), rabbit anti-JNK (1:1000, Proteintech, 24164-1-AP), rabbit anti-E-cadherin (1:1000, Proteintech, 20874-1-AP), rabbit anti-Vimentin (1:1000, Proteintech, 10366-1-AP), rabbit anti-ZEB1 (1:1000, Proteintech, 21544-1-AP), rabbit anti-RAC1 (1:1000, Proteintech, 24072-1-AP), rabbit anti-CDC42 (1:1000, Proteintech, 10155-1-AP), and mouse anti-glyceraldehyde 3-phosphate dehydrogenase (1:1000, Abcam, ab8245) antibodies overnight at 4 °C. Subsequently, they were incubated with horseradish peroxidase (HRP)-labeled rabbit IgG secondary antibodies (1:2500, Invitrogen, #31460) and HRP-labeled mouse IgG secondary antibodies (1:2500, Invitrogen, #31430), and the protein bands were visualized using the ChemiDoc TM MP Imaging System (BioRad).

### Immunohistochemistry analysis

Immunohistochemical (IHC) staining was conducted using PowerVision™ Two-Step Histostaining Reagent (Zhongshan Golden Bridge Biotechnology, Beijing, China). Briefly, after deparaffinization and blocking, the slices were incubated with rabbit anti-DTL (1:150, Abcam), anti-FOXO1 (1:100, Cell Signaling Technology), anti-E-cadherin, and anti-Vimentin (1:200, Proteintech) antibodies for 12 h at 4 °C in a wet chamber. Then the slides were incubated with goat anti-rabbit IgG for 30 min, stained with diaminobenzidine (ZSGB-BIO, ZLI9018) for 2 min, and counterstained with hematoxylin, dehydrated, and mounted. Each stained slide was independently assessed by two pathologists in a double-blinded manner.

### Cell Counting Kit 8 assay

Cell viability was measured using Cell Counting Kit 8 (Dojindo Laboratories, Kumamoto, Japan). Exponentially growing cells were plated in 96-well plates and measured at optical density 450 nm with the BioTek Gen5 system (BioTek, Winooski, VT, USA) every 24 h over the next 5 days.

### Wound-healing assay

Cell migration was assessed by measuring the movement of the cells. Cells were plated in six-well plates until they achieved 95% confluency; subsequently, scratch wounds were created in each well using a 200 µL pipette tip and recorded at 0 and 24 h.

### Transwell assays

The transwell invasive and migration assays were performed in 24-well plates with transwell inserts (Corning, MA, USA) with or without pre-coated Matrigel. Cells were seeded at a density of 5 × 10^4^ cells per upper well in a 200 µL culture medium containing 2% FBS, with the lower chambers containing 500 µL of medium added with 20% FBS. After 24 h, the upper surface was gently removed using a cotton swab and the lower surface was fixed with methanol and stained with hematoxylin and eosin (H&E). Four fields of view were randomly selected for quantification under the microscope.

### In vivo metastasis assay

Four to five-week-old female BALB/c-nude mice were acquired from the Beijing Vital River Laboratory Animal Technology Co., Ltd (Beijing, China), and were housed under standard conditions. Mice were randomly divided into two groups (*n* = 5 mice in each group) and two groups of mice were administered intravenous caudal vein injections of 1 × 10^6^ cells (SiHa-Vec/SiHa-DTL and Caski-Vec/Caski-DTL) per mouse, single-blind experiment. All mice were killed after receiving tail vein injections for 6 weeks. Cryosections (4 mm) were used for H&E and IHC staining. All steps were approved by the Harbin Medical University Institutional Animal Care and Use Committee.

### Statistical analysis

All statistical analyses were performed using R software (version 3.6.1) (http://www.R-project.org/) or GraphPad Prism 8 (GraphPad Software, Inc., La Jolla, CA, USA). R packages, such as limma, ggplot2, and pheatmap, were used to generate figures. The *χ*^2^-test and Student’s *t*-test test were used to analyze the differences between the two groups. Overall survival curves were obtained using the Kaplan–Meier method and compared using the log-rank test. All tests were two-tailed and *P*-values < 0.05 were considered statistically significant. Quantitative data are expressed as mean ± SD.

## Results

### DTL is highly expressed in cervical adenocarcinoma

**Fig. 1:**
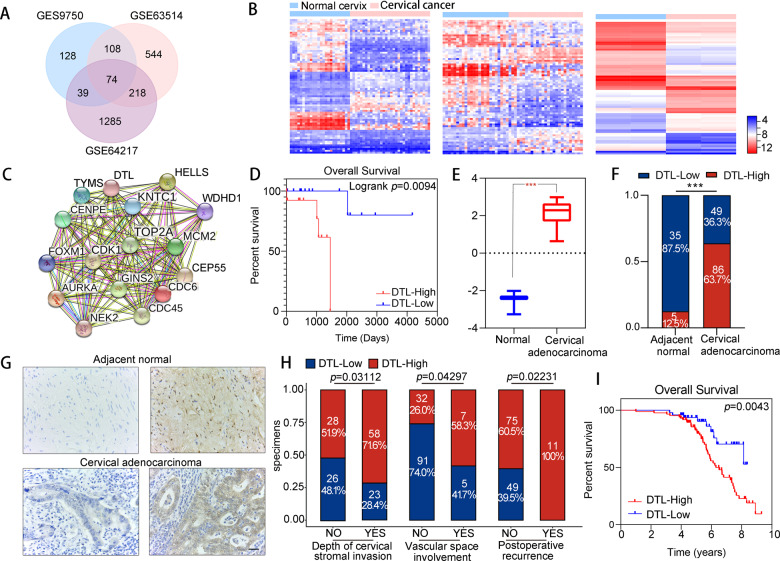
Expression of DTL in cervical cancer data sets and its prognostic importance in patients with cervical adenocarcinoma. **A** Venn diagrams of DEGs in three gene expression data sets. **B** Heatmaps generated using the 74 common DEGs among the three data sets. **C** Significant module in the PPI network constructed from STRING using the common DEGs with MCODE scores = 15.467. **D** Overall survival of patients with cervical adenocarcinoma through the TCGA database (*n* = 28, *P* = 0.0094, log-rank test). **E** Boxplot of DTL expression in normal and cervical adenocarcinoma groups of TCGA samples. **F**, **G** Staining rate of DTL and representative images of DTL immunochemistry in adjacent normal tissue and cervical adenocarcinoma tissue (magnification, ×400). **H** DTL expression was significantly interrelated with the depth of cervical stromal invasion, vascular space involvement, and postoperative recurrence. **I** Overall survival of patients with cervical adenocarcinoma was analyzed using Kaplan–Meier curves. Each patient was classified as having “low” or “high” DTL expression (*n* = 135, log-rank test). Error bars indicate the mean ± SD of three independent experiments. Student’s *t*-test; *P < 0.05, **P < 0.01, ***P < 0.001.

Data sets GSE64217, GSE63514, and GSE9750 were retrieved from the GEO database. Cervical cancer tissues were compared with normal tissues for analysis, using *P* < 0.01 and |logFC| > 2 as the cutoff criteria. It was found that 74 genes were expressed differently in all the three data sets (Fig. 1A, B). MCODE was used to analyze and detect 16 hub genes (Fig. 1C). Among these 16 hub genes, only the expression level of DTL could be used as a predictor of survival in patients with cervical adenocarcinoma through the TCGA database (*P* = 0.0094, log-rank test) (Fig. 1D and Supplementary Fig. 1). Therefore, DTL was selected for further study. Along with the normal group, DTL was also highly expressed in cervical adenocarcinoma tissues through the TCGA database (Fig. 1E).

To explore the expression status of DTL in cervical adenocarcinoma, pathological specimens of 135 patients with cervical adenocarcinoma and 40 normal specimens that contained clinical data were analyzed by IHC staining. The positive staining rate of DTL was significantly higher in cervical cancer tissues (86 of 135 [63.7%]) than in non-tumor tissues (5 of 40 [12.5%]) (*P* < 0.001) (Fig. 1F, G). In addition, DTL overexpression was associated with the depth of invasion of the cervical stroma (*P* = 0.03112), vascular space involvement (*P* = 0.04297), and postoperative recurrence (*P* = 0.02231) among patients with cervical adenocarcinoma (Fig. 1H and Table 1). Patients with cervical adenocarcinoma with high DTL expression showed significantly worse overall survival (*P* = 0.0043, log-rank test) (Fig. 1I). These results showed that DTL is highly expressed in cervical adenocarcinoma and is associated with tumor invasion and poor patient prognosis.

**Table 1:** Interrelation between DTL expression and clinicopathological features of cervical adenocarcinoma tissues.

Clinical parameters		DTL expression		*p*-Value^a^
	No. of patients (*N* = 135)	Normal, no. (%) (*N* = 49, 36.3%)	Overexpression, no. (%) (*N* = 86, 63.7%)	
Age (years)				0.05887
>47	64	29 (45.3)	35 (54.7)	
≥47	71	20 (28.2)	51 (71.8)	
FIGO stage				0.9073
I	97	36 (37.1)	61 (62.9)	
II	38	13 (34.2)	25 (65.8)	
Tumor size (mm)				1
≤40	121	44 (36.4)	77 (63.6)	
>40	14	5 (35.7)	9 (64.3)	
Depth of cervical stromal invasion				**0.03112**
<1/2	54	26 (48.1)	28 (51.9)	
≥1/2	81	23 (28.4)	58 (71.6)	
Surgical margin				1
Negative	134	49 (36.6)	85 (63.4)	
Positive	1	0 (0.0)	1 (100.0)	
Lymphovascular space invasion				0.1231
No	114	45 (39.5)	69 (60.5)	
Yes	21	4 (19.0)	17 (81.0)	
Pelvic lymph node metastasis				0.08046
No	116	46 (39.7)	70 (60.3)	
Yes	19	3 (15.8)	16 (84.2)	
Parametrium invasion				0.7378
Negative	133	49 (36.8)	84 (63.2)	
Positive	2	0 (0.0)	2 (100.0)	
Vascular space involvement				**0.04297**
No	123	91 (74.0)	32 (26.0)	
Yes	12	5 (41.7)	7 (58.3)	
Postoperative recurrence				**0.02231**
No	124	49 (39.5)	75 (60.5)	
Yes	11	0 (0.0)	11 (100.0)	

*DTL* denticleless E3 ubiquitin protein ligase homolog, *FIGO* International Federation of Gynecology and Obstetrics.

Significant *P* < 0.05 are shown in bold.

^a^*χ*^2^-test.

### DTL overexpression promotes cell migration and invasion, and induces EMT in cells

**Fig. 2:**
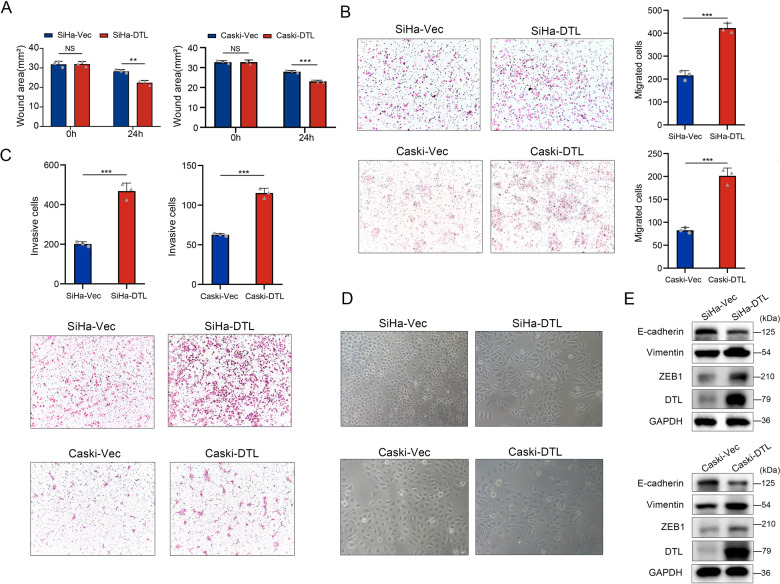
DTL enhanced tumor cell migration and invasion in vitro and induced EMT. **A** Wound-healing assay showing that DTL overexpression enhanced the migration of SiHa and Caski cells. **B**, **C** Transwell assays indicating that DTL overexpression stimulated cell migration (**B**) and invasion (**C**) in SiHa and Caski cells. **D**, **E** Cell images were taken after stably expressing vector or DTL (**D**) and western blotting was performed (**E**). Error bars indicate the mean ± SD of three independent experiments. Student’s *t*-test; *P < 0.05, **P < 0.01, ***P < 0.001.

To investigate the impact of DTL on the biological characteristics of cervical cancer cells, three cell lines (SiHa, Caski, and Hela) were selected to detect the expression levels of DTL by western blotting. We observed that DTL was weakly expressed in SiHa and Caski cells (Supplementary Fig. 2A). For overexpression study, we constructed SiHa and Caski stable cell lines and verified them by western blotting (Supplementary Fig. 2B). Wound-healing assay showed that the cells that overexpressed DTL showed improved healing ability (*P* < 0.01, Fig. 2A and Supplementary Fig. 2D). In the transwell assays, cells that overexpressed DTL showed greater migration (*P* < 0.001, Fig. 2B) and invasion abilities (*P* < 0.001, Fig. 2C) than the control cells did. However, no significant change was observed in their proliferation ability (Supplementary Fig. 2C).

We also observed that DTL-transfected SiHa and Caski cells appeared as long spindles under the microscope (Fig. 2D). We speculate that this may have been caused by EMT. To investigate this, we analyzed the expression levels of EMT markers and found that the expression of Vimentin was increased, whereas that of E-cadherin was decreased by western blotting. We noticed that the EMT-activator ZEB1 was increased as well (Fig. 2E). This result further confirmed that DTL promoted EMT, and invasion and metastasis of cervical cancer cells.

### FOXO1 participates in DTL-induced EMT, and migration and invasion of tumor cells

**Fig. 3:**
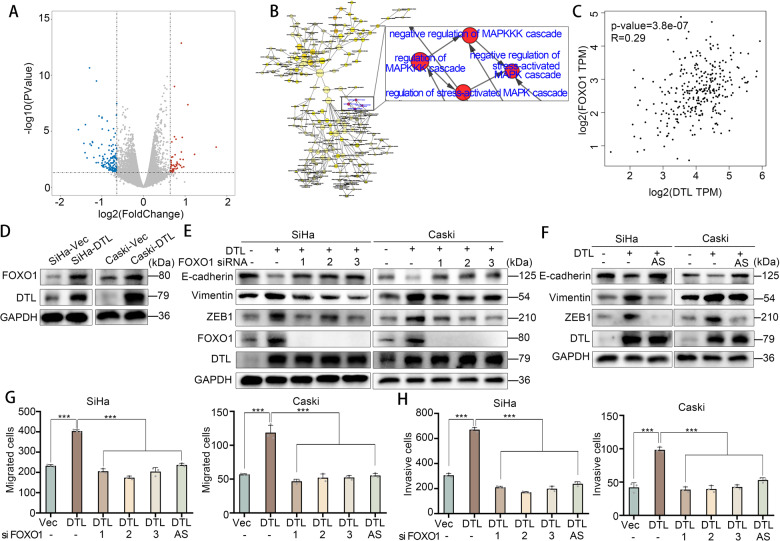
DTL-induced EMT and cell migration and invasion through FOXO1. **A** Heatmap showing the top 200 up- or downregulated genes affected by DTL overexpression. **B** Enriched pathways for the top 200 genes associated with DTL in RNA-Seq assay including “SAPK/JNK pathway.” **C** FOXO1 expression is positively correlated with DTL through the TCGA database. **D** Western blotting demonstrated that DTL overexpression increases FOXO1 protein levels. **E**, **F** FOXO1 siRNAs (**E**) or FOXO1 inhibitor (AS1842856) (**F**) reduces DTL-induced EMT. **G**, **H** Quantified data of transwell migration (**G**) or invasion (**H**) assay in SiHa and Caski cells. Error bars indicate the mean ± SD of three independent experiments. Student’s *t*-test; *P < 0.05, **P < 0.01, ***P < 0.001.

To further study the role of DTL in the migration and invasion of cervical cancer cells, SiHa-Vec and SiHa- DTL cells were subjected to RNA sequencing. The volcano plot shows the top 200 up- or downregulated genes, using *P* < 0.05 and |logFC| > 0.64 as the cutoff criteria (Fig. 3A). We entered these genes into BiNGO and observed the enrichment in mitogen-activated protein kinase pathways, including SAPK/JNK pathway (*P* < 0.01, Fig. 3B). Among the upregulated and downregulated genes, FOXO1 is a well-known suppressor that promotes the migration and invasion of tumor cells [14–16]. We also observed that FOXO1 expression is positively associated with DTL, through the TCGA database (Fig. 3C). Therefore, we sought to explore the involvement of FOXO1 in DTL-induced EMT.

Western blotting revealed that DTL overexpression increased the level of FOXO1 (Fig. 3D). Knockdown of FOXO1 led to suppression of DTL-induced EMT (Fig. 3E). Using a FOXO1 inhibitor, AS1842856, we confirmed that FOXO1 inhibition suppressed DTL-induced EMT as well (Fig. 3F). Similarly, knockdown of FOXO1 or cells with FOXO1 inhibitor inhibited cell migration (*P* < 0.001, Fig. 3G and Supplementary Fig. 3A) and invasion (*P* < 0.001, Fig. 3H and Supplementary Fig. 3B) induced by DTL. These results demonstrate that FOXO1 plays an essential role in DTL-induced EMT. In addition, FOXO1 is indispensable for DTL-induced cell migration and invasion.

### DTL induces FOXO1 upregulation and is regulated by JNK

**Fig. 4:**
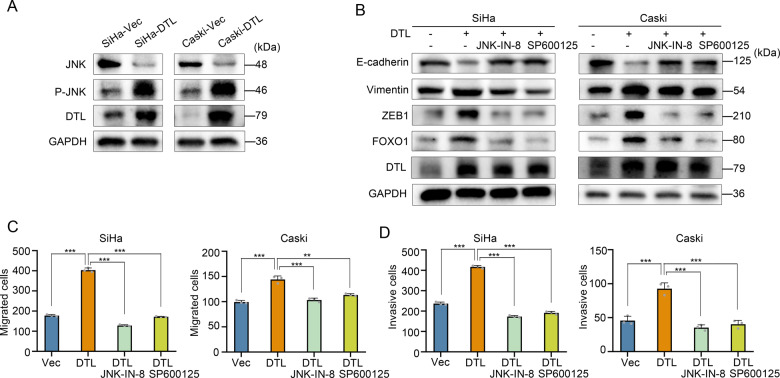
JNK mediates DTL-induced EMT, FOXO1 upregulation, cell migration, and invasion. **A** Western blotting showed that DTL overexpression induced JNK phosphorylation. **B** DTL-induced EMT was suppressed by JNK inhibitor (JNK-IN-8 and SP600125). **C**, **D** Quantified data of transwell migration (**C**) or invasion (**D**) assay in SiHa and Caski cells. Error bars indicate the mean ± SD of three independent experiments. Student’s *t*-test; *P < 0.05, **P < 0.01, ***P < 0.001.

In the previous enrichment analysis, we found that the SAPK/JNK pathway was affected by DTL overexpression (Fig. 3B). Therefore, we conducted further experiments and observed that DTL overexpression induced JNK phosphorylation (Fig. 4A). As FOXO1 can be regulated by direct modifiers such as JNK, p38, and LRKK2 [14], we found that use of JNK inhibitors significantly reduced the upregulation of FOXO1 expression caused by DTL overexpression; moreover, inhibition of JNK suppressed DTL-induced EMT (Fig. 4B). Similarly, JNK inhibition significantly suppressed DTL-induced migration (*P* < 0.001, Fig. 4C and Supplementary Fig. 4A) and invasion (*P* < 0.001, Fig. 4D and Supplementary Fig. 4B) of cervical cancer cells. Further, knockdown experiments were performed on DTL overexpression by DTL siRNA transfection, which showed that DTL-induced phosphorylation of JNK and DTL-induced EMT were lost, and the expression level of FOXO1 was reduced (Supplementary Fig. 4C). These results showed that the activation of JNK by DTL increased FOXO1 level and EMT induction, and promoted cell migration and invasion.

### DTL regulates JNK and EMT through the RAC1 pathway

**Fig. 5:**
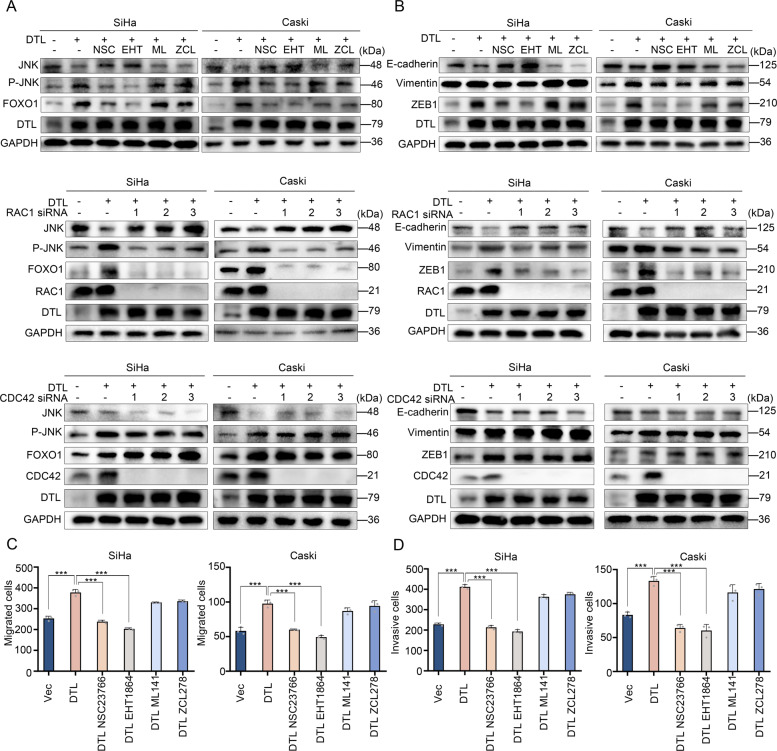
RAC1-JNK pathway is related to DTL-induced cell migration, invasion, and EMT. **A**, **B** Western blotting showed that DTL-induced upregulation of FOXO1, JNK phosphorylation (**A**), and EMT (**B**) was regulated by RAC1 but not CDC42. **C**, **D** Quantified data of transwell migration (**C**) or invasion (**D**) assay in SiHa and Caski cells. Error bars indicate the mean ± SD of three independent experiments. Student’s *t*-test; *P < 0.05, **P < 0.01, ***P < 0.001.

The JNK pathway is activated through mechanisms majorly mediated by GTPases including RAC1 and CDC42 [17]. To determine whether these activators participate in DTL-mediated JNK activation, we performed the following experiments. We observed that RAC1 inhibitors markedly reduced DTL-induced phosphorylation of JNK and upregulation of FOXO1 but not inhibition of CDC42. Knockdown of RAC1 and CDC42 yielded similar results (Fig. 5A). DTL-induced EMT was significantly suppressed when RAC1, but not CDC42, was inhibited or knocked down (Fig. 5B). This finding was also confirmed in the migration (*P* < 0.001, Fig. 5C and Supplementary Fig. 5A) and Matrigel invasion assays (*P* < 0.001, Fig. 5D and Supplementary Fig. 5B). These findings showed that RAC1 played a critical role in DTL-induced FOXO1 upregulation, JNK phosphorylation, and EMT.

### DTL promotes metastasis in vivo

**Fig. 6:**
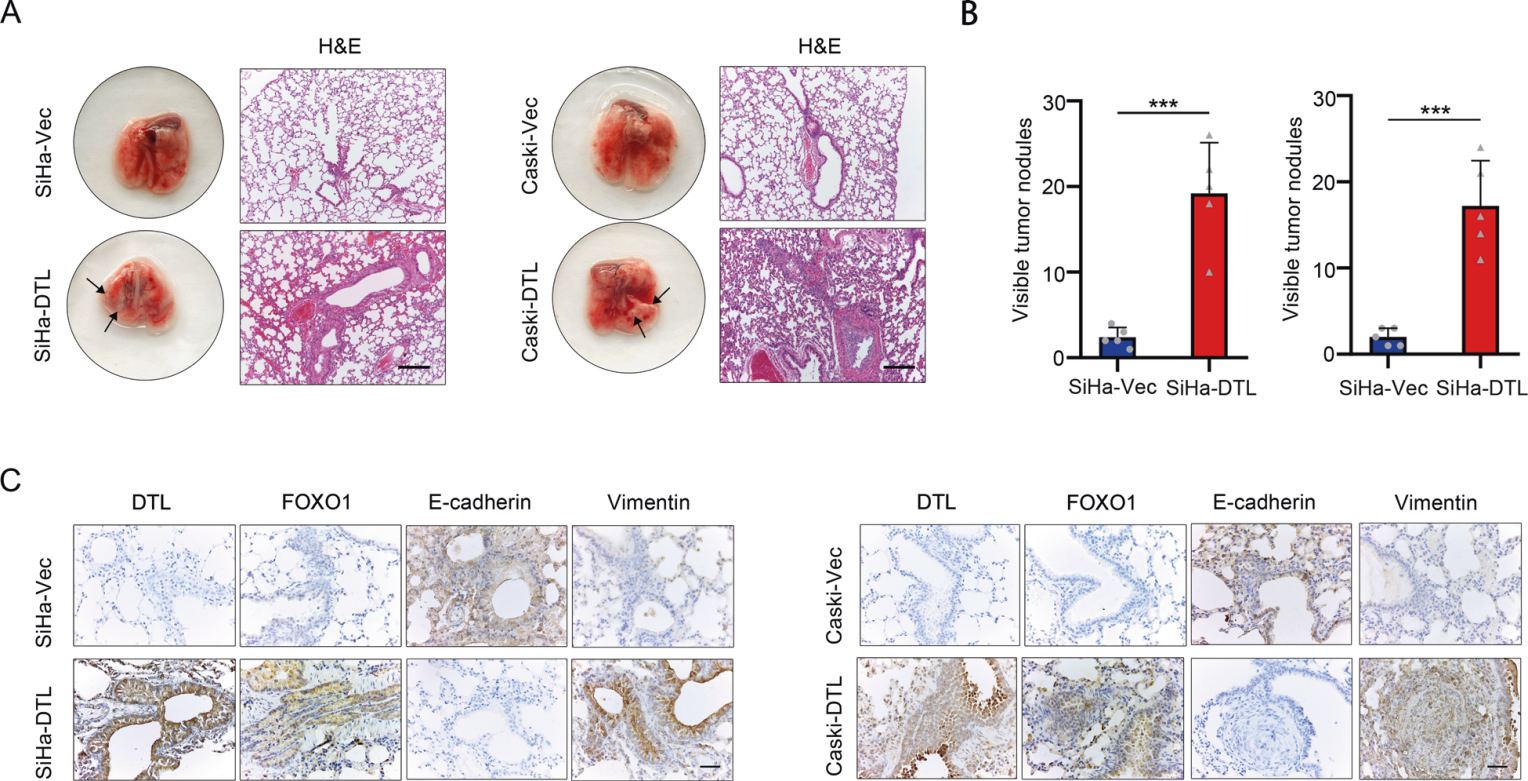
DTL enhanced the metastatic potential of tumor cells in vivo. **A** H&E staining and representative images of lung metastatic nodules in a mouse model (magnification, ×100). **B** Quantified data of lung metastatic nodules. **C** Sections of lung metastatic nodules were subjected to IHC staining. Error bars indicate the mean ± SD of three independent experiments (magnification, ×400). Student’s *t*-test; *P < 0.05, **P < 0.01, ***P < 0.001.

Although DTL overexpression promoted the invasion and migration of cells in vitro, we extended our research to explore whether DTL could induce tumor metastasis in vivo. Thus, we induced DTL overexpression and intravenously injected control cervical cancer cells in the tail vein of 5-week-old female nude mice (*n* = 5 mice per group). Six weeks after injection, we found that more metastatic nodules were formed on the lung surfaces of nude mice injected with SiHa-DTL and Caski-DTL cells, confirmed by H&E staining (*P* < 0.01, independent Student’s *t*-test, Fig. 6A, B). Mice with DTL overexpression showed increased levels of FOXO1 and EMT marker Vimentin, which was verified by IHC staining. Moreover, the mice with DTL overexpression showed reduced levels of E-cadherin (Fig. 6C).

## Discussion

In the current study, we found that DTL overexpression promoted the migration and invasion of cervical cancer cells, leading to adverse prognosis of patients with cervical adenocarcinoma. Further, the overexpression of DTL utilized the RAC1-JNK-FOXO1 axis to promote cell migration, cell invasion, and EMT. Our study showed that DTL promotes the migration and invasion of tumor cells in cervical adenocarcinoma via the JNK-FOXO1 axis.

The incidence of cervical adenocarcinoma has been increasing in recent years [2] and this disease has a poor prognosis. The main factors influencing the prognosis of cervical adenocarcinoma are invasion and metastasis [3–5]. In our study, we observed that high expression of DTL affects vascular space involvement, depth of cervical stromal invasion, postoperative recurrence, and overall survival of patients with cervical adenocarcinoma. In particular, DTL overexpression encourages the invasion and migration of cervical cancer cells. EMT has a significant role in tumor progression, especially metastasis [18]. The EMT-activator ZEB1 is highly relevant to the regulation of the ubiquitination process, tumor invasion, and metastasis in cancers [19–23]. In our study, overexpression of DTL induced the progression of EMT and RNA sequencing was performed to clarify the specific mechanisms. FOXO1 plays an active role in cancer cell migration, invasion, and metastasis in specific cancers or diseases [14–16]. In this study, FOXO1 expression was upregulated, owing to increased expression of DTL. We also found that FOXO1 inhibition suppressed DTL-induced EMT and reduced the invasion and migration of cervical cancer cells. RNA-sequencing data suggested that increased DTL expression also changed the expression of the JNK pathway. JNK pathway is essential for the progression and preservation of phenotypic and cellular changes related to EMT [24]. FOXO1 expression can be regulated by direct modifiers such as JNK, p38, and LRKK2 [14]. We observed that inhibition of JNK suppressed DTL-induced EMT and reduced the upregulation of FOXO1 expression. The JNK pathway is activated through mechanisms majorly mediated by GTPases including RAC1 and CDC42 [17]. Our research showed that only RAC1 and not CDC42 could regulate DTL-induced JNK/FOXO1 signal axis and EMT. In addition, we are investigating the mechanism of activation of the RAC1-JNK-FOXO1 axis by DTL. Our hypothesis tentatively suggests that DTL activates JNK by directly interacting with the COP9 signalosome complex.

Most tumor cells do not undergo complete EMT but acquire some qualities of mesenchymal cells and retain some epithelial characteristics [18, 25]. The change in E-cadherin levels in our study could be attributed to the ubiquitination of DTL. A previous study has reported similar results, stating that E3 ubiquitin ligase Hakai ubiquitinates E-cadherin, which is related to the regulation of EMT during development or metastasis [10]. FOXO1 is a widely known tumor suppressor, owing to its role as a tumor motility inhibitor and tumor death inducer [26]. Unlike the current speculation that FOXOs can inhibit tumors, our research suggested that FOXO1 activated by DTL could increase the migration and invasion potential of tumor cells by inducing EMT, which exhibits a tumor-promoting effect. Recent evidence suggests that the functions of several cancer modulators depend on various conditions and cellular environments [27–29]. In addition, FOXO1 actively participates in the migration, invasion, and metastasis of cancer cells in specific cancers or diseases [15, 16]. The results of these studies provide sufficient basis for our research. Although the incidence of cervical adenocarcinoma is increasing, it accounts for 20% of all cervical cancers [30], rendering it difficult to collect data. In this study, we collected the data of 135 patients with early-stage cervical adenocarcinoma. The large amount of data provided a sufficient clinical basis for this research.

Studies have shown that DTL may serve as a prognostic marker for cancers [31–33]. Our research showed that the overall survival of patients with cervical adenocarcinoma was associated with the expression of DTL. Our findings not only reveal the specific mechanism by which DTL promotes EMT, but also shows the potential for DTL to serve as a prognostic marker of cervical adenocarcinoma.

Our findings also provide a potential theoretical basis for cervical adenocarcinoma cells to be more prone to invasion and migration. EMT is closely related to the sensitivity of cancer cells to radiotherapy [34, 35]. Next, we plan to study the effect of DTL on the sensitivity of cervical adenocarcinoma cells to radiotherapy and assess its molecular mechanism, so as to provide new targets for individualized treatment of patients with cervical adenocarcinoma.

## Supplementary information

Author Contribution Form

Supplementary figure 1

Supplementary figure 2

Supplementary figure 3

Supplementary figure 4

Supplementary figure 5

Author Contribution Form
