# Supplementary material for: Overexpression of DTL enhances cell motility and promotes tumor metastasis in cervical adenocarcinoma by inducing RAC1-JNK-FOXO1 axis
Source: Cell Death Dis. 2021 Oct 11;12(10):929. doi: 10.1038/s41419-021-04179-5 (PMC8505428; doi:10.1038/s41419-021-04179-5)
Supplement: Supplementary file 1 — Author Contribution Form [file 41419_2021_4179_MOESM1_ESM.docx]

**Supplementary figure legends**

**Fig. S1** Effect of expression levels of another 15 hub genes on overall survival of patients with cervical adenocarcinoma through the TCGA database (n = 28, log-rank test)

**Fig. S2** DTL induced cell migration and invasion but not proliferation

(A) Western blotting showing endogenous DTL expression status in three cervical cancer cell lines; (B) Transfection of DTL in SiHa and Caski cells; (C) CCK-8 assay demonstrated that overexpression of DTL has no significant effect on cell proliferation (Student’s *t*-test); and (D) Typical images of wound healing assay

**Fig. S3** DTL-induced cell migration and invasion was mainly mediated by FOXO1

(A–B) Representative images of transwell migration (A) or invasion (B) assay in SiHa and Caski cells with or without FOXO1 siRNAs or FOXO1 inhibitor (AS1842856)

**Fig. S4** JNK mediates DTL-induced cell migration and invasion

(A–B) Representative images of transwell migration (A) or invasion (B) assay in SiHa and Caski cells with or without JNK inhibitors (JNK-IN-8 and SP600125); (C) Western blotting showing that knockdown of DTL in DTL-overexpressing cells suppressed DTL-induced phosphorylation of JNK and DTL-induced EMT, and reduced FOXO1 protein levels

**Fig. S5** DTL-induced cell migration and invasion was regulated by RAC1 but not CDC42

(A–B) Representative images of transwell migration (A) or invasion (B) assay in SiHa and Caski cells stably expressing vector control or DTL with or without RAC1 or CDC42 inhibitors
