# Supplementary figures and images for: Overexpression of DTL enhances cell motility and promotes tumor metastasis in cervical adenocarcinoma by inducing RAC1-JNK-FOXO1 axis
Source: Cell Death Dis. 2021 Oct 11;12(10):929. doi: 10.1038/s41419-021-04179-5 (PMC8505428; doi:10.1038/s41419-021-04179-5)

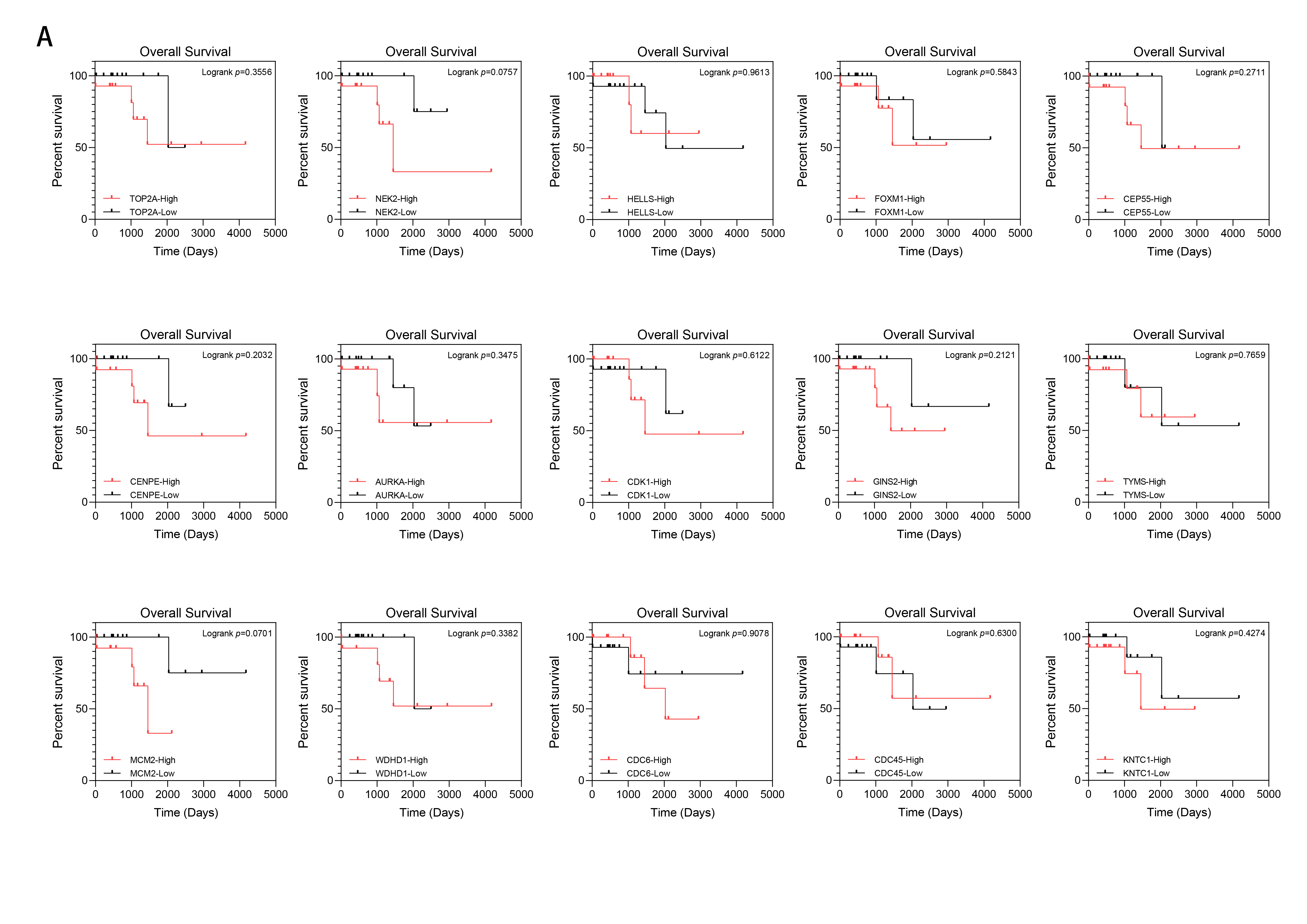

Supplement: Supplementary file 2 — Supplementary figure 1 [file 41419_2021_4179_MOESM2_ESM.jpg]

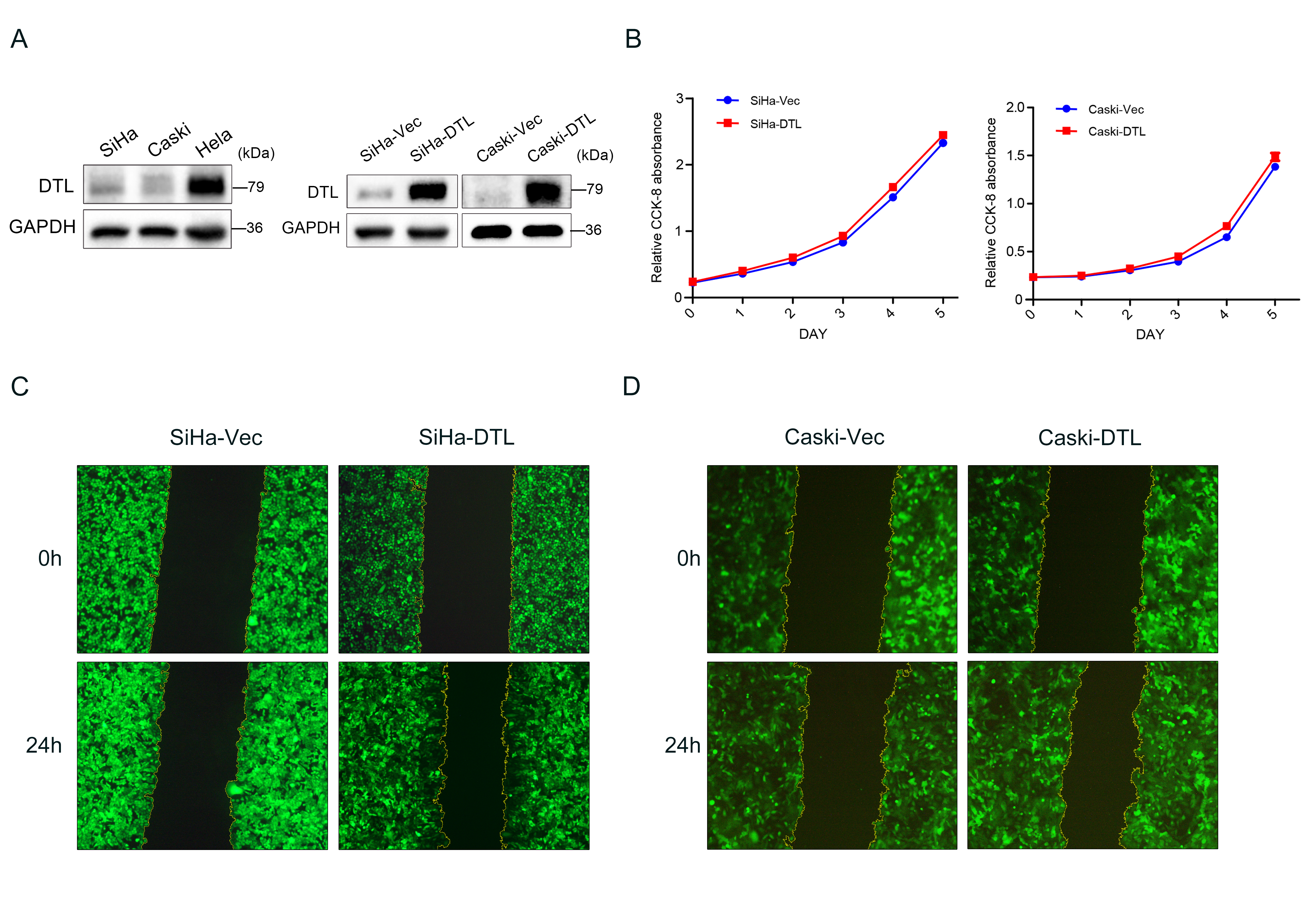

Supplement: Supplementary file 3 — Supplementary figure 2 [file 41419_2021_4179_MOESM3_ESM.jpg]

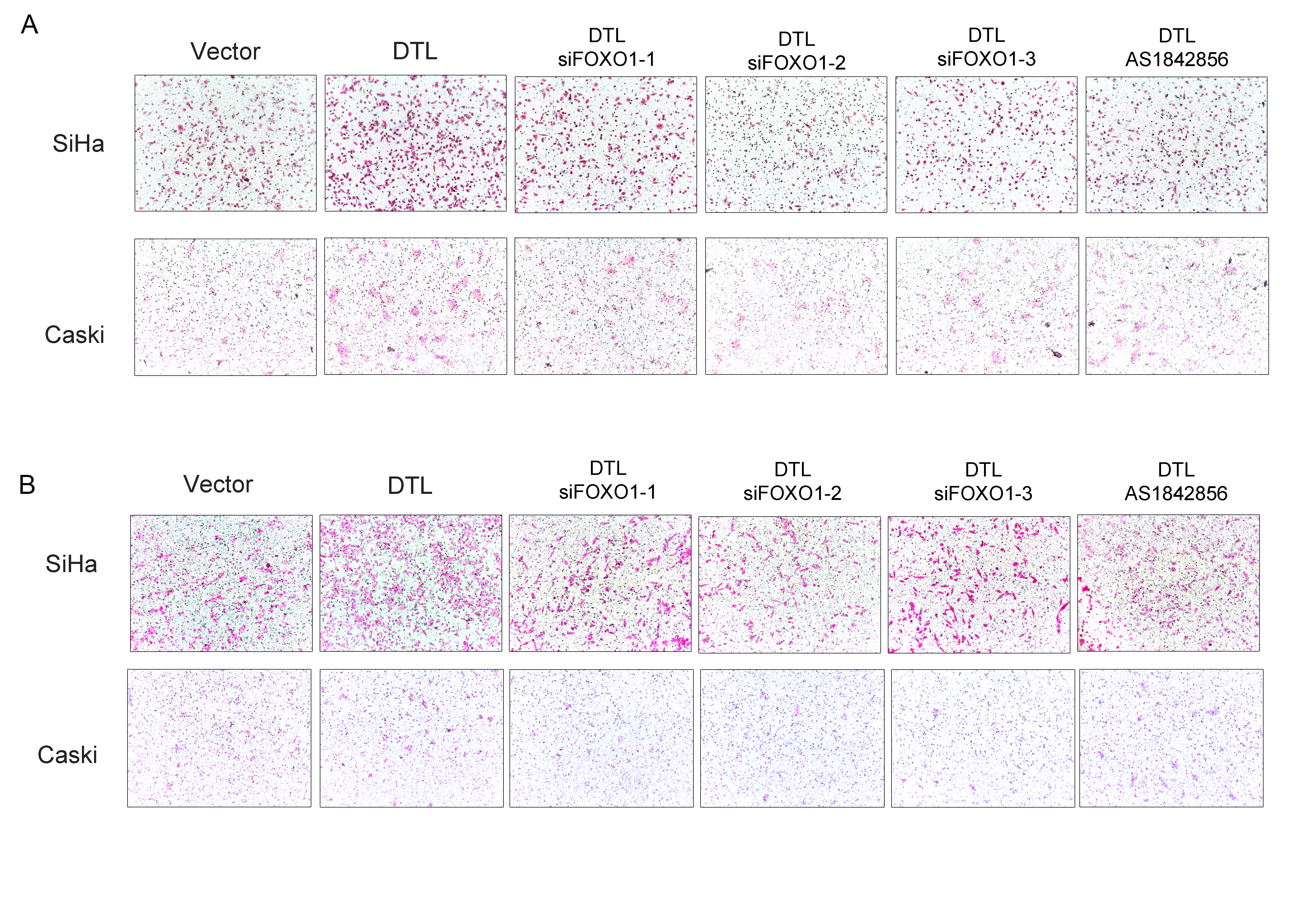

Supplement: Supplementary file 4 — Supplementary figure 3 [file 41419_2021_4179_MOESM4_ESM.jpg]

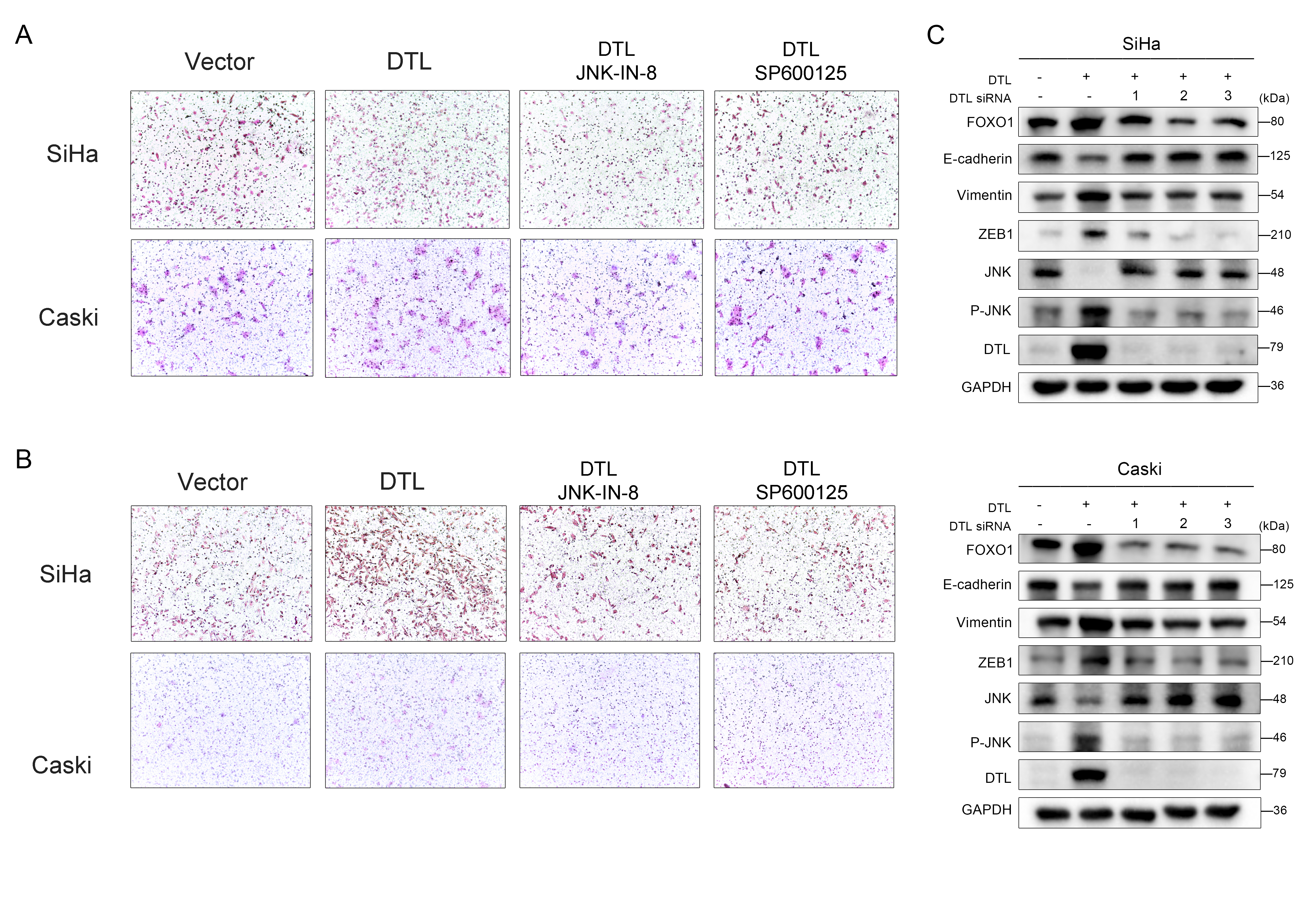

Supplement: Supplementary file 5 — Supplementary figure 4 [file 41419_2021_4179_MOESM5_ESM.jpg]

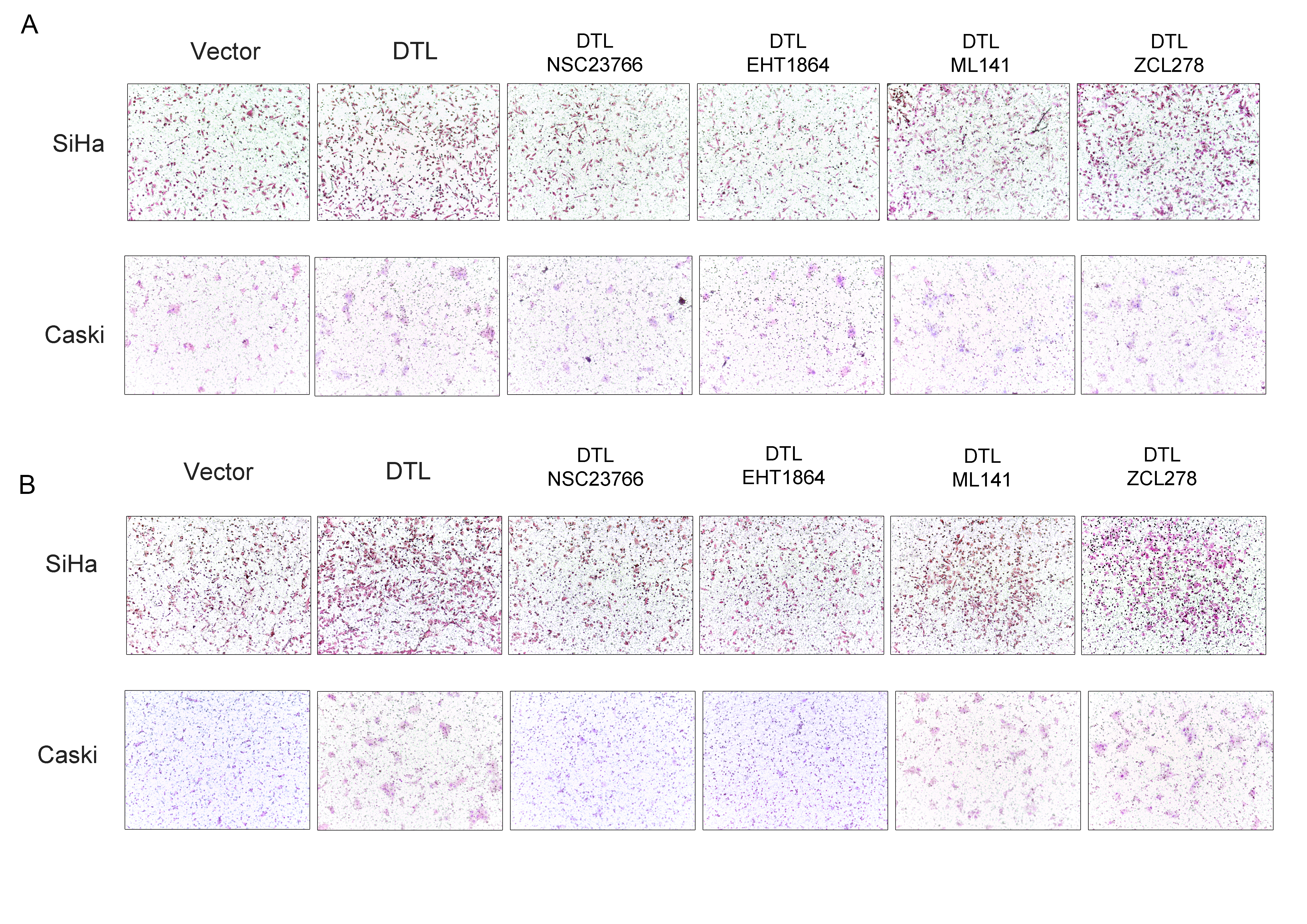

Supplement: Supplementary file 6 — Supplementary figure 5 [file 41419_2021_4179_MOESM6_ESM.jpg]
