# Supplementary material for: Overexpression of DTL enhances cell motility and promotes tumor metastasis in cervical adenocarcinoma by inducing RAC1-JNK-FOXO1 axis
Source: Cell Death Dis. 2021 Oct 11;12(10):929. doi: 10.1038/s41419-021-04179-5 (PMC8505428; doi:10.1038/s41419-021-04179-5)
Supplement: Supplementary file 7 — Author Contribution Form [file 41419_2021_4179_MOESM7_ESM.pdf]

DECLARATION OF CONTRIBUTIONS TO ARTICLE

ADMC

Manuscript Number:	Journal Name:	
	Cell Death & Disease	(the 'Journal')

Proposed Title of the Contribution:

Overexpression of DTL Enhances Cell Motility and Promotes Tumor Metastasis in Cervical Adenocarcinoma by Inducing RAC1-JNK-FOXO1 Axis (the 'Contribution)

Author(s):

Sijia Liu, Lina Gu, Nan Wu, Jiayu Song, Jiazhuo Yan, Shanshan Yang, Yue Feng, Zhao Wang, Le Wang, Yunyan Zhang, and Yan Jin (the 'Authors')

For all *CDDis* articles, each person named as an author in the published version must be able to show he or she has contributed substantially to the article.

Authorship credit should be based on 1) substantial contributions to conception and design, acquisition of data, or analysis and interpretation of data; 2) drafting the article or revising it critically for important intellectual content; and 3) final approval of the version to be published. Authors should meet conditions 1, 2 and 3.

Any person who cannot be shown to have made a substantial contribution to the article cannot be listed as an author in the final version. The name of any person who is deemed to have made a minor contribution can, however, appear in the Acknowledgments section of the article.

Please complete the table below to indicate the contributions of all named authors to the manuscript.

Author Full Name:	Specification of Contribution to the Manuscript:
Sijia Liu	devised the project and the main conceptual ideas;formulated most of the technical details;performed the experiment;wrote the manuscript
Lina Gu	collected clinical data;substantial contributions to conception and design
Nan Wu	formulated most of the technical details;performed the experiment
Jiayu Song	performed the experiment
Jiazhuo Yan	verified the numerical results by an independent implementation
Shanshan Yang	substantial contributions to conception and design
Yue Feng	collected clinical data
Zhao Wang	collected clinical data
Le Wang	substantial contributions to conception and design
Yunyan Zhang	devised the project and the main conceptual ideas;formulated most of the technical details;editorial changes to the manuscript
Yan Jin	devised the project and the main conceptual ideas

Please complete the table below to indicate the contributions of all named authors to the figures.

Figure 1:
Sijia Liu,Lina Gu,Yue Feng,Zhao Wang,Jiayu Song,Le Wang,Yunyan Zhang,Yan Jin

Figure 2:
Sijia Liu,Nan Wu,Jiayu Song,Shanshan Yang,Lina Gu,Yunyan Zhang,Yan Jin

Figure 3:
Sijia Liu,Nan Wu,Jiazhuo Yan,Shanshan Yang,Jiayu Song,Yunyan Zhang,Yan Jin

Figure 4:
Sijia Liu,Nan Wu,Jiazhuo Yan,Shanshan Yang,Yunyan Zhang,Yan Jin

Figure 5:
Sijia Liu,Nan Wu,Jiazhuo Yan,Shanshan Yang,Yunyan Zhang,Yan Jin

Figure 6:
Sijia Liu,Lina Gu,Nan Wu,Yue Feng,Zhao Wang,Le Wang,Yunyan Zhang,Yan Jin

Signed for and on behalf of the Author(s):	Print Name:	Date:
Yunyan Zhang	Yunyan Zhang	2021/4/15
